# Patient-reported Outcome Measures and Decision Regret After Prostate-specific Membrane Antigen–targeted Radioguided Surgery for Oligorecurrent Prostate Cancer

**DOI:** 10.1016/j.euros.2024.09.010

**Published:** 2024-10-10

**Authors:** Fabian Falkenbach, Giovanni Mazzucato, Zhe Tian, Pierre I. Karakiewicz, Markus Graefen, Thomas Steuber, Derya Tilki, Daniel Koehler, Burkhard Beyer, Pierre Tennstedt, Sophie Knipper, Lars Budäus, Tobias Maurer

**Affiliations:** a Martini-Klinik Prostate Cancer Center, University Medical Center Hamburg-Eppendorf, Hamburg, Germany; b Cancer Prognostics and Health Outcomes Unit, Division of Urology, University of Montréal Health Center, Montréal, Québec, Canada; c Department of Urology, University of Verona, Azienda Ospedaliera Universitaria Integrata, Verona, Italy; d Department of Urology, University Medical Center Hamburg-Eppendorf, Hamburg, Germany; e Department of Diagnostic and Interventional Radiology and Nuclear Medicine, University Medical Center Hamburg-Eppendorf, Hamburg, Germany; f Urological Competence Center for Rehabilitation Wildetal, Bad Wildungen, Germany; g Department of Urology, Vivantes Klinikum am Urban, Berlin, Germany

**Keywords:** Salvage surgery, Biochemical recurrence, Prostate-specific membrane antigen positron emission tomography, Imaging-guided surgery, Quality of life, Patient-reported outcome measures

## Abstract

**Background and objective:**

In patients with oligorecurrent prostate cancer (PCa), prostate-specific membrane antigen–targeted radioguided surgery (PSMA-RGS) prolongs treatment-free survival. Data on patient-reported outcome measures (PROMs) are lacking.

**Methods:**

A retrospective assessment of validated PROMs (12-item Short Form Health Survey [SF-12], 26-item Expanded Prostate Index Composite, and Decision Regret Scale [DRS]) was performed before and after PSMA-RGS for oligorecurrent PCa. Mixed models were used.

**Key findings and limitations:**

A total of 373 patients were analyzed at a median (interquartile range [IQR]) age of 66 (61, 70) yr and prostate-specific antigen of 0.8 (0.4, 1.5) ng/ml. Six months after PSMA-RGS, the median (IQR) scores for the PROMs were as follows: SF-12 physical 54 (49, 56), SF-12 mental 53 (43, 56), urinary incontinence 86 (52, 100), urinary irritation 94 (88, 100), sexual 27 (9, 57), hormonal 90 (79, 100), and bowel 96 (83, 100). Only the sexual score decreased in a significant fashion from baseline over time (median [IQR], 17 [8,38]) after 3 yr vs 37 [13, 63] at baseline, *p* = 0.01). The decision regret remained low (median [IQR] DRS at 1 yr: 5 [0, 20]). More than 90% of the patients reported that PSMA-RGS was the correct decision after 1 yr.

**Conclusions and clinical Implications:**

We recorded no significant decrease in quality of life or any functional status domain, except sexual. While decision regret was low, sexual functioning might deteriorate further.

**Patient summary:**

No significant deterioration in health-related quality of life was reported after removing early prostate cancer metastases. Very few patients expressed remorse about their decision for salvage surgery.

## Take Home Message

In patients with oligorecurrent prostate cancer after radical prostatectomy, prostate-specific membrane antigen–targeted radioguided surgery (PSMA-RGS) may prolong treatment-free survival without negatively affecting the quality of life or any functional status domains, except sexual. More than 90% of the patients reported that PSMA-RGS was the correct decision after 1 yr.

## 1 Introduction

The timing and treatment modality of early oligorecurrent prostate cancer (PCa) that is visible only on prostate-specific membrane antigen (PSMA) imaging remains a matter of debate [1], [2], [3]. Metastasis-directed therapy (MDT) delays the necessity of systemic treatment [4], [5], [6], [7] and its associated consequences on quality of life (QoL) and general health [8], [9]. Deterioration of hormonal and sexual domains of patient-reported outcome measures (PROMs) has been reported for androgen-deprivation therapy (ADT) [10], as well as for its combination with novel androgen receptor pathway inhibitors (ARPIs) [11], [12]. Hot flushes, emotional lability, depression, fatigue, changes in sexual performance, and body feminization reduce the QoL in men treated with ADT significantly [8], [13].

However, the impact of surgical MDT on PROMs has not yet been assessed, although many men contemplate MDT as an alternative to immediate ADT for oligorecurrent PCa. We addressed this knowledge gap in a contemporary cohort of PCa patients with limited soft-tissue/nodal recurrence after radical prostatectomy (RP), who were treated with PSMA-targeted radioguided surgery (PSMA-RGS).

## 2 Patients and methods

### 2.1 Study population

We included all consecutive patients treated with PSMA-RGS at our center between 2018 and 2023. Of the 376 patients, three (1%) were excluded from further analysis because of initial local therapy other than RP (two patients) and castration-resistant PCa (one patient). All patients presented with one or more soft-tissue or lymph-node lesion(s) on PSMA imaging after initial RP, with or without adjuvant/salvage radiotherapy (RT). Written consent was obtained for all procedures, data collection, and analysis. This retrospective study was approved by the local review board (2019-PS-09; PV7316). All data were stored prospectively in an institutional database (FileMaker; Claris Inc., Cupertino, CA, USA).

### 2.2 Procedure of MDT using PSMA-RGS

The PSMA-RGS procedure has been reported previously [14], [15], [16]. In summary, patients were injected with 99m-technetium–labeled, PSMA-targeting imaging and therapy agents, resulting in increased gamma emission of cells with PSMA expression. The following day, regional excision of local recurrences or template-based lymph-node dissection for nodal recurrences was performed using gamma-probe measurements to facilitate detection and resection of cancerous tissue.

### 2.3 Patient-reported outcome measures

Validated PROMs were collected prior to as well as 1 and 6 mo after PSMA-RGS, and then annually. The validated 12-item Short Form Health Survey (SF-12) measured general health-related QoL subdivided by physical and mental scores [17], [18], [19]. The disease-specific functional impact of PSMA-RGS was measured with the validated 26-item Expanded Prostate Index Composite (EPIC-26), with the following domain-specific function summary scores: urinary incontinence, urinary irritative, sexual, hormonal, and bowel [12]. Changes in each EPIC-26 domain were considered clinically meaningful if the validated minimally important differences (MIDs) were met: urinary incontinence 6–9, urinary irritative 5–7, sexual 10–12, hormonal 4–6, and bowel 4–6 [20]. For the functional and QoL scores, a higher value was indicative of better function and QoL. Distress or remorse was measured using the 5-item Decision Regret Scale (DRS) [21], [22]. A higher DRS score represents increased regret. PROMs were collected using the International Consortium for Health Outcomes Measurement [23] certified Questlink platform (Philips, Amsterdam, The Netherlands) in English [24] or in a validated German translation [25], [26]. Severe complications were defined as any Clavien-Dindo ≥IIIb complication. The questionnaire data were not censored for subsequent treatments unless stated otherwise.

### 2.4 Statistical analyses

Differences were assessed using the chi-square test (categorical variables) or Wilcoxon rank-sum test (continuous variables). We calculated the significance of differences between baseline and postoperative time points, assuming that the missing data at baseline were missing at random. The findings derived from these assumptions were validated using a subgroup analysis of patients with baseline and postoperative measurements only. Linear mixed-effect models (R packages: lme4 and lmerTest) were used to identify potential correlations between clinical parameters and PROMs after PSMA-RGS measured repeatedly over time at the patient level (conditional growth model with removing the random slope). For treatment-free survival estimation, the Kaplan-Meier method was used. All statistical analyses were performed using R version 4.3.1 (R Foundation for Statistical Computing, Vienna, Austria) [27]. All tests were two sided, with the level of significance set at *p* < 0.05.

## 3 Results

### 3.1 Patient and surgery characteristics

Overall, follow-up information was available for 365/373 (98%) patients, and 304/373 (82%) patients completed PROM questionnaires. The median (interquartile range [IQR]) follow-up for patients was 26 (13, 42) mo. At PSMA-RGS, the median (IQR) age and prostate-specific antigen (PSA) was 66 (61, 70) yr and 0.8 (0.4, 1.5) ng/ml, respectively (Table 1). The median (IQR) time between RP and PSMA-RGS was 46 (23, 85) mo. Of 373 patients, 24 (6%) received neoadjuvant ADT 6 mo prior to PSMA-RGS. A robot-assisted approach for PSMA-RGS was used in 48/373 (13%) patients. Severe complications were observed in 26/373 (7%) patients (Supplementary Table 1). Cancerous tissue could be removed in 352/373 (94%) patients. A complete biochemical response (cBR, postoperative PSA <0.2 ng/ml) was achieved in 207/373 (56%) patients. Of 373 patients, 132 (35%) reported further PCa treatment at a median (95% confidence interval [CI]) of 38 (31, not available) mo. Of these, most patients received ADT (91/132, 69%) or RT (32/132, 24%) as the next treatment. The estimated (95% CI) treatment-free survival rates after 1 and 2 yr were 78% (73%, 82%) and 60% (54%, 66%), respectively. The proportion of PROM questionnaire responders was higher among patients who achieved a cBR (183/207, 88% vs 121/166, 74%; *p* < 0.01) and consecutively in patients with lower PSA prior PSMA-RGS (median [IQR], 0.7 [0.6,2.3] vs 1.0 [0.4,1.4] ng/ml; *p* < 0.01). There were no statistically significant differences in other baseline characteristics at PSMA-RGS, such as age or surgical approach, between questionnaire responders and nonresponders (*p* > 0.05).

**Table 1:** Descriptive characteristics of patients undergoing prostate-specific membrane antigen–targeted radioguided surgery for oligorecurrent prostate cancer

	All patients (*n* = 373)
Age at PSMA-RGS (yr), median (IQR)	66 (61, 70)
Radiotherapy after RP, *n* (%)	195 (52)
PSA at PSMA-RGS (ng/ml), median (IQR)	0.8 (0.4, 1.5)
Robot-assisted approach, *n* (%)	48 (13)
Severe complications a*n* (%)	26 (7)
Complete biochemical response b*n* (%)	207 (56)
Positive pathology report (cancerous tissue removed), *n* (%)	352 (94)
Treatment-free survival estimate at 1 yr c % (95% CI)	78 (73, 82)
Treatment-free survival estimate at 2 yr c % (95% CI)	60 (54, 66)
Patients with PROMs d*n* (%)	304 (82)

CI = confidence interval; IQR = interquartile range; PROMs = patient-reported outcome measures; PSA = prostate specific antigen; PSMA-RGS = prostate-specific membrane antigen–targeted radioguided surgery; RP = radical prostatectomy.

a Clavien-Dindo ≥IIIb.

b Complete biochemical response was defined as postoperative PSA <0.2 ng/ml without any additional treatment.

c Kaplan-Meier point estimate for 1 yr after salvage surgery.

d Patients who completed at least one PROM questionnaire.

### 3.2 General health-related QoL

**Fig. 1:**
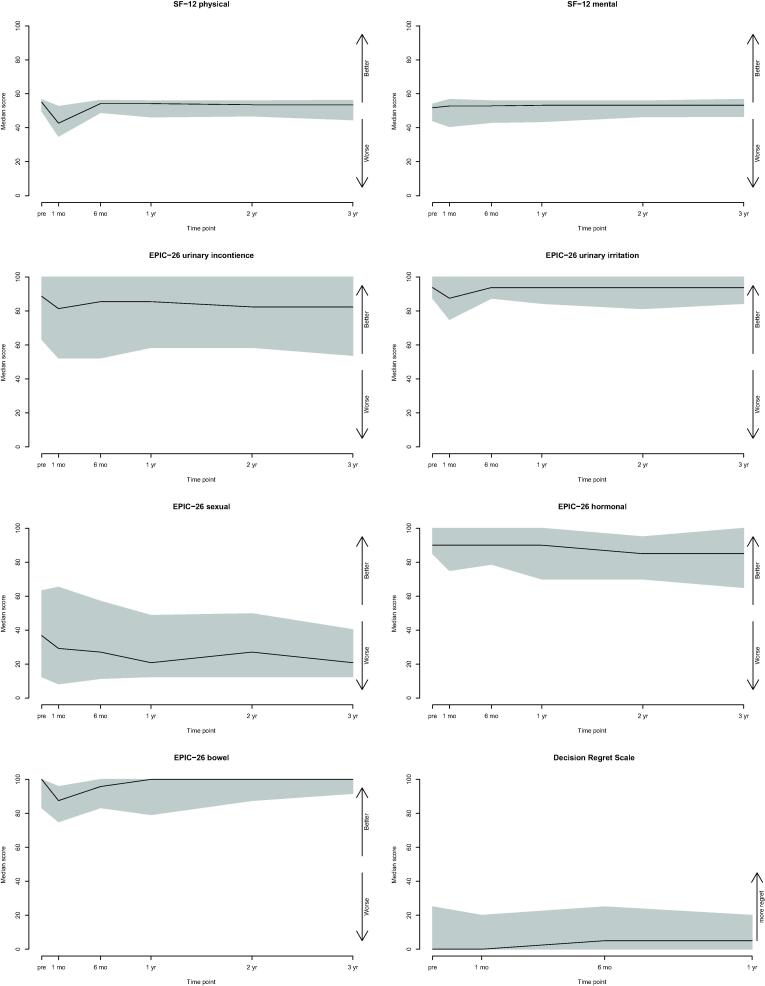
Patient-reported functional status, quality of life, and decision regret after PSMA-targeted radioguided surgery for oligorecurrent prostate cancer. The time point is measured relative to the PSMA-RGS procedure, that is, before and 1 yr after PSMA-RGS. The black line shows the median score, and the gray area shows the interquartile range at each time point. For SF-12 and EPIC-26, higher scores indicate better quality of life and functional status. For DRS, higher scores represent more regret. DRS = Decision Regret Scale; EPIC-26 = 26-item Expanded Prostate Index Composite; Pre = before PSMA-RGS; PSMA-RGS = prostate-specific membrane antigen–targeted radioguided surgery; SF-12 = 12-item Short Form Health Survey.

Prior to PSMA-RGS, the median (IQR) SF-12 physical and mental health scores were 55 (50, 57) and 52 (44, 54) (Table 2), respectively. One month after PSMA-RGS, the median (IQR) SF-12 physical score was significantly lower at 43 (35, 53) (*p* < 0.001) and returned to baseline levels after 6 mo (54 [49, 56]; *p* = 0.4). The SF-12 mental health score after 6 mo was 53 (41, 57) and did not change significantly over time (Fig. 1). In a mixed model analysis (MMA), advanced age at PSMA-RGS and robot-assisted approach were correlated significantly with increased SF-12 mental health score (*p* ≤ 0.01).

**Table 2:** Patient-reported outcome measures of patients undergoing prostate-specific membrane antigen–targeted radioguided surgery for oligorecurrent prostate cancer a, b

Domain/time point	Baseline	After 1 mo	After 6 mo	After 1 yr
SF-12 physical	55 (50, 57)	**43 (35, 53)**	54 (49, 56)	54 (46, 56)
SF-12 mental	52 (44, 54)	53 (41, 57)	53 (43, 56)	53 (44, 56)
EPIC-26 urinary incontinence	89 (63, 100)	81 (52, 100)	86 (52, 100)	86 (59, 100)
EPIC-26 urinary irritation	94 (88, 100)	88 (75, 100)	94 (88, 100)	94 (84, 100)
EPIC-26 sexual	37 (13, 63)	29 (8,65)	27 (9, 57)	18 (13, 49)
EPIC-26 hormonal	90 (85, 100)	90 (75, 100)	90 (79, 100)	90 (70, 100)
EPIC-26 bowel	100 (83, 100)	**88 (75, 96)**	96 (83, 100)	100 (79, 100)
Decision Regret Scale	0 (0, 25)	0 (0, 20)	5 (0, 25)	5 (0, 20)

EPIC-26 = 26-item Expanded Prostate Index Composite; PSMA-RGS = prostate-specific membrane antigen–targeted radioguided surgery; SF-12 = 12-item Short Form Health Survey.

a Data are presented as median (interquartile range).

b Time points were measured relative to the PSMA-RGS. Bold values indicate statistically significant change compared with baseline level (*p* < 0.05).

### 3.3 Impacts on functional domains

The median (IQR) EPIC-26 urinary incontinence score was 89 (63, 100) at baseline, and decreased to 81 (52, 100) at 1 mo and 86 (52, 100) at 6 mo after PSMA-RGS. This change was neither statistically significant nor clinically meaningful according to the MID criteria. The number of patients reporting the use of more than one pad per day did not change significantly 1 yr after surgery compared with baseline (3% vs 2%, *p* = 0.6). In the MMA, advanced age at PSMA-RGS was correlated significantly with decreased EPIC-26 urinary incontinence scores (*p* = 0.03).

The median (IQR) EPIC-26 urinary irritation score was 94 (88, 100) at baseline, but decreased to 88 (75, 100) at 1 mo and recovered to 94 (88, 100) at 6 mo after PSMA-RGS. Although the initial change might be considered borderline clinically meaningful, further changes from baseline were neither statistically significant nor clinically meaningful according to the MID criteria. In the MMA, RT before PSMA-RGS was correlated significantly with decreased EPIC-26 urinary obstructive scores (*p* = 0.03).

The median (IQR) EPIC-26 sexual score was 37 (13, 63) at baseline, and decreased to 29 (8, 65) at 1 mo, 27 (9,57) at 6 mo, 18 (13,49) at 1 yr, and 17 (8,38) at 3 yr after PSMA-RGS. While the initial decrease did not reach statistical significance or the criterion for a clinically meaningful decrease, a clear downward tendency was observed and the deterioration fulfilled the MID criteria at 1 yr. The rate of men reporting erections firm enough for intercourse was 13% after 1 yr (compared with 14% at baseline, *p* > 0.05). If questionnaires were censored at subsequent ADT, the median (IQR) EPIC-26 sexual score was 27 (12, 57) at 6 mo, 21 (13, 49) at 1 yr, and 21 (13, 40) at 3 yr (Supplementary Fig. 1). In the MMA, advanced age before PSMA-RGS and an open surgical approach were correlated significantly with decreased EPIC-26 sexual scores (*p* < 0.01). In fact, no correlation with prior ADT was observed (*p* = 0.09).

The median (IQR) EPIC-26 hormonal score was 90 (85, 100) at baseline, and remained unchanged at 90 (75,100) at 1 mo and at 90 (79, 100) at 6 mo after PSMA-RGS. This change was not statistically significant or clinically meaningful. After 3 yr, a statistically significant and clinically meaningful decrease compared with baseline was recorded (at 3 yr: 85 [65, 100]; *p* = 0.04). However, if questionnaires were censored at subsequent ADT, no significant change in the hormonal score was recorded (at 3 yr: 90 [74, 100], *p* = 0.9). In the MMA, not achieving a cBR was correlated significantly with decreased EPIC-26 hormonal scores (*p* < 0.05).

The median (IQR) EPIC-26 bowel score was 100 (83, 100) at baseline, but decreased to 88 (75, 96) at 1 mo and recovered to 96 (83, 100) at 6 mo after PSMA-RGS. Although the initial decrease was statistically significant (*p* < 0.001) and clinically meaningful (–12 points), further changes from baseline were not. In the MMA, RT prior to PSMA-RGS correlated significantly with decreased EPIC-26 bowel scores (*p* = 0.01).

### 3.4 Decision regret

After 1 yr, the median (IQR) DRS score was 5 (0, 20), indicating low or no relevant decision regret (DR). More than 90% of the patients reported that PSMA-RGS was the correct decision 1 yr later. In the MMA, cancerous tissue removed, cBR, and absence of severe complications postoperatively were significantly correlated with less regret in the DRS (*p* < 0.05). In the MMA, neoadjuvant ADT was not significantly correlated with more regret in the DRS (*p* = 0.5).

## 4 Discussion

The widespread use of PSMA imaging for biochemical recurrence has led to increased detection of early oligorecurrent PCa, in which MDT alone is able to delay disease progression and the need for systemic therapy. Given the current oncological uncertainties in this situation, informed shared decision-making necessitates an understanding of future patients’ perceptions on health-related functional status and QoL after salvage surgery with PSMA-RGS. Therefore, our analyses revealed several noteworthy findings with direct clinical implications.

First, the overall impact of PSMA-RGS on functional and QoL PROMs after 6 mo was marginal. Most detriments occurred early and resolved promptly, as was also observed in primary PCa treatments [28], [29]. We recorded no significant decrease in SF-12 QoL (physical/mental) or any EPIC-26 domains at 6 mo or later. The changes in any domain except sexual did not meet the validated MID criteria for clinical meaningfulness. In contrast, significant and continuous deterioration of hormonal and sexual function has been described for ADT ± ARPI, which constitutes the most common treatment alternative [10], [11], [12], [30]. For instance, in the TOAD trial, immediate versus delayed ADT for biochemical recurrence decreased hormonal and sexual function permanently in a clinically meaningful and statistically significant fashion, whereas global QoL and other functional domains did not differ [10]. Hormonal symptoms such as hot flushes, sore breasts/nipples, and general feeling of reduced virility are among the common fears of men contemplating ADT for, after all, asymptomatic PSA/PSMA imaging–only recurrence. An intermittent ADT schedule may lessen hormonal treatment–related symptoms, but testosterone levels need a median of 10 mo to recover, limiting the benefit of intermittent ADT [31]. Modern (combination) therapies, such as, enzalutamide ± ADT, have shown similar or, in combination, increased rates of hormonal treatment–related symptoms [11], [12]. Our results also compare favorably with studies on salvage RT in which all EPIC-26 domains worsened significantly [32]. Therefore, our results are reassuring for men seeking a treatment alternative without hormonal treatment–related detriments and with otherwise negligible influences on functional status and QoL.

Second, the sexual function score decreased gradually from 37 before to 18 1 yr after PSMA-RGS. However, the effect was less pronounced if patients with subsequent ADT were censored, and the low baseline sexual function must be acknowledged. Only one out of seven men was able to maintain an erection sufficient for intercourse prior to PSMA-RGS. Functional deterioration due to subsequent ADT and aging may exaggerate the effects of the PSMA-RGS itself. Thus, also in several active surveillance cohorts, patients reported a gradual decline in sexual function over time [28], [33], [34]. We also observed a statistically significant correlation between advanced age at PSMA-RGS and a decrease in EPIC-26 sexual scores. Moreover, any other treatment modality exposes men in this situation to a high risk of further loss of sexual function as well [35]. Despite all efforts, erectile dysfunction might be seen as the natural fate of PCa survivors in the long run. Fifteen years after local therapy, 87% (RP) and 94% (RT) of men reported an inability to achieve an erection sufficient for intercourse [36]. Taken together, low sexual function at baseline, natural deterioration by aging, and the lack of treatment modalities without an impact on sexual functioning put the observed changes into perspective and limit the overall pertinence in decision-making.

Third, DR was low and inversely associated with oncological and procedural success. Overall, >90% of the patients did not regret the decision to undergo salvage surgery after 1 yr, and the corresponding median DRS score was 5. While the rates of DR and its measurement methods vary widely [37], these are excellent results for procedures that merely delay further treatment in most cases. This might partly be explained by the fact that the common alternative ADT is associated with the highest rates of DR [38], [39]. Accordingly, failing to remove cancerous tissue or achieve a cBR was associated with an increased DRS score over time. Conversely, the absence of severe complications was correlated with less regret, as also seen in studies on RP [37].

Fourth, the baseline scores prior to PSMA-RGS were comparable with those of other contemporary studies assessing PROMs after RP [28], [29], [33], [40]. For instance, the CEASAR trial reported, 3 yr after nerve-sparing RP, median [IQR] scores of 79 (58, 100) for urinary incontinence (vs 89 [63, 100] at baseline in our study), 94 (88, 100) for urinary irritation (vs 94 [88, 100]), 48 (14, 79) for sexual (vs 37 [13, 63]), 100 (96, 100) for bowel (vs 100 [83, 100]), and 95 (85, 100) for hormonal (vs 90 [85, 100]) (33). Notably, in non–nerve-sparing RP patients, a median (IQR) score for sexual function of 20 (0, 53) was reported. Moreover, the established risk factors from the primary setting were confirmed in the PSMA-RGS setting. Age was correlated with increased urinary incontinence and decreased sexual functioning. Age is an established risk factor for urinary incontinence and decreased sexual function after RP [29], [36]. RT prior to PSMA-RGS correlated with decreased bowel and urinary irritation scores, also known from the primary situation [28], [29], [30], [36]. This cross-study comparison validates the applied methodology and suggests the generalizability of our results.

Despite the novelty of our study, several limitations must be acknowledged. First, confounding by indication is likely, and patients with low functional and QoL status may undergo PSMA-RGS at a lower rate. Second, there is no consensus on which PROMs and analytical methods should be considered the gold standard. In particular, handling of missing data differed significantly across trials. Third, the EPIC-26 focuses on the aspect of sexual function, that is, the erectile function (to penetrate). It does not cover all biopsychosocial dimensions of sexual health, including desire, orgasm quality, and psychosocial effects.

## 5 Conclusions

Our analyses empower men to make informed decisions regarding the tradeoff between potential oncological benefits, and the functional and QoL costs of surgical MDT. Although DR is low and QoL is not affected significantly, sexual functioning might deteriorate further.

  ***Author contributions*:** Tobias Maurer had full access to all the data in the study and takes responsibility for the integrity of the data and the accuracy of the data analysis.

  *Study concept and design*: Falkenbach, Maurer.

*Acquisition of data*: Beyer, Mazzucato, Knipper, Koehler, Beyer.

*Analysis and interpretation of data*: Falkenbach, Tian, Tennstedt.

*Drafting of the manuscript*: Falkenbach, Maurer.

*Critical revision of the manuscript for important intellectual content*: Karakiewicz, Graefen, Steuber, Tilki, Koehler, Beyer, Knipper, Budäus, Maurer.

*Statistical analysis*: Falkenbach, Tian, Tennstedt.

*Obtaining funding*: None.

*Administrative, technical, or material support*: None.

*Supervision*: Maurer, Graefen.

*Other*: None.

  ***Financial disclosures:*** Tobias Maurer certifies that all conflicts of interest, including specific financial interests and relationships and affiliations relevant to the subject matter or materials discussed in the manuscript (eg, employment/affiliation, grants or funding, consultancies, honoraria, stock ownership or options, expert testimony, royalties, or patents filed, received, or pending), are the following: None.

  ***Funding/Support and role of the sponsor*:** None.

  ***Ethics statement*:** This study was conducted in accordance with the ethical standards of the institutional and national research committee and with the 1964 Declaration of Helsinki and its later amendments, and was approved by the local institutional review board. All patients included in the study provided written informed consent for the procedure and data analysis.
