# Supplementary material for: Patient-reported Outcome Measures and Decision Regret After Prostate-specific Membrane Antigen–targeted Radioguided Surgery for Oligorecurrent Prostate Cancer
Source: Eur Urol Open Sci. 2024 Oct 10;70:1–7. doi: 10.1016/j.euros.2024.09.010 (PMC11736159; doi:10.1016/j.euros.2024.09.010)
Supplement: Supplementary Data 1 [file mmc1.docx]

**Supplementary Material**

|  | **Severe Complications^1^ *n = 26 (7%)*** |
| --- | --- |
| **Ureter injury,** *n* | 6 |
| **Bleeding complications****,** *n* | 5 |
| **Bowel injury,** *n* | 5 |
| **Lymphocele with intervention,** *n* | 2 |
| **Urethral stricture,** *n* | 3 |
| **Hernia,** *n* | 2 |
| **Impaired wound healing****,** *n* | 2 |
| **Death,** *n* | 1 |
| **^1^Clavien-Dindo ≥IIIb.** | |

**Supplementary Table 1: Severe complications** **of patients undergoing prostate-specific membrane antigen–targeted radioguided surgery for oligorecurrent prostate cancer.**

|  | **Overall *n = 373*** |
| --- | --- |
| **Presacral lesion,** *n [%]* | 51 [14] |
| **Retrovesical lesion,** *n [%]* | 58 [16] |
| **Other pelvic** **lesion^†^,** *n [%]* | 253 [68] |
| **Retroperitoneal lesion,** *n [%]* | 69 [18] |
| *† The majority of cases are lesions within the pelvic lymph nodes alongside the obturator, external iliac, internal iliac, or common iliac area.*  *Number do not add up because patients may harbor more than one lesion.* | |

**Supplementary Table 2: Location of lesions on PSMA-imaging of patients undergoing prostate-specific membrane antigen–targeted radioguided surgery for oligorecurrent prostate cancer.**

**Supplementary Figure 1: Patient-reported sexual functional status after PSMA–targeted radioguided surgery for oligorecurrent prostate cancer, censored at the moment of initiation of androgen-deprivation therapy.**

The time-point is measured relative to the PSMA-RGS procedure, i.e., "pre" = before PSMA-RGS, "1 yr" = one year after PSMA-RGS. The black line shows the median score and the gray area shows the interquartile range at each time point. Higher scores indicate better functional status.
